# Epstein-Barr Virus-Induced Gene 3 (EBI3) Blocking Leads to Induce Antitumor Cytotoxic T Lymphocyte Response and Suppress Tumor Growth in Colorectal Cancer by Bidirectional Reciprocal-Regulation STAT3 Signaling Pathway

**DOI:** 10.1155/2016/3214105

**Published:** 2016-05-10

**Authors:** Yanfang Liang, Qianqian Chen, Wenjing Du, Can Chen, Feifei Li, Jingying Yang, Jianyu Peng, Dongping Kang, Bihua Lin, Xingxing Chai, Keyuan Zhou, Jincheng Zeng

**Affiliations:** ^1^Department of Pathology, Dongguan Hospital, Medical College of Jinan University, The Fifth People's Hospital of Dongguan, Dongguan 523905, China; ^2^Guangdong Provincial Key Laboratory of Medical Molecular Diagnostics, Guangdong Medical University, Dongguan 523808, China; ^3^Department of Integrative Medicine, Huashan Hospital, Fudan University, Shanghai 200040, China

## Abstract

Epstein-Barr virus-induced gene 3 (EBI3) is a member of the interleukin-12 (IL-12) family structural subunit and can form a heterodimer with IL-27p28 and IL-12p35 subunit to build IL-27 and IL-35, respectively. However, IL-27 stimulates whereas IL-35 inhibits antitumor T cell responses. To date, little is known about the role of EBI3 in tumor microenvironment. In this study, firstly we assessed EBI3, IL-27p28, IL-12p35, gp130, and p-STAT3 expression with clinicopathological parameters of colorectal cancer (CRC) tissues; then we evaluated the antitumor T cell responses and tumor growth with a EBI3 blocking peptide. We found that elevated EBI3 may be associated with IL-12p35, gp130, and p-STAT3 to promote CRC progression. EBI3 blocking peptide promoted antitumor cytotoxic T lymphocyte (CTL) response by inducing Granzyme B, IFN-*γ* production, and p-STAT3 expression and inhibited CRC cell proliferation and tumor growth to associate with suppressing gp130 and p-STAT3 expression. Taken together, these results suggest that EBI3 may mediate a bidirectional reciprocal-regulation STAT3 signaling pathway to assist the tumor escape immune surveillance in CRC.

## 1. Introduction

The Epstein-Barr virus-induced gene 3 (EBI3) is a member of the interleukin-12 (IL-12) family structurally homologous to the IL-12p40 subunit and forms a heterodimer either with the IL-27p28 subunit to build IL-27 or with IL-12p35 subunit to form IL-35. IL-27 is secreted by activated antigen presenting cells with an antitumor via promoting cytotoxic T lymphocyte (CTL) [1–3] and NK cells function [4, 5], regulating T cell subsets differentiation [6], suppressing DC function [3], and angiogenesis [7]. However, IL-35 appears to be produced mainly by regulatory T or B cells (Treg or Breg) and epithelial derived tumor cells with a protumor via expanding Tregs and inhibiting CD4^+^CD25^−^ effector T cells [8], promoting IL-35-producing CD1d^high^CD5^+^ B cells mediated tumor cell proliferation [9], enhancing myeloid cell accumulation [10], and inhibiting tumor cell apoptosis [11, 12]. Our previous reports also found tumor-derived IL-35 or EBI3 associated with IL-12p35 may recruit Treg cells into the tumor microenvironment in favor of tumor growth in human colorectal cancer (CRC) [13]. However, the exact mechanisms of EBI3 in CRC tumor microenvironment are not fully understood.

In this work we further tested the role of EBI3 in tumor microenvironment with a EBI3 blocking peptide (EBI3 Bp). To this aim, we firstly evaluated the relationship of EBI3, IL-27p28, IL-12p35, gp130, and p-STAT3 expression with clinicopathological parameters on 50 CRC tissues. Then, we isolated CRC cells and tumor infiltrating lymphocytes (TIL) from 10 CRC patients tissues to assess the Granzyme B, IFN-*γ*, EBI3, IL-27p28, and IL-12p35 production and proliferation of TILs, Tregs after coculture with autologous CRC cells blocked by EBI3 blocking peptide. Meanwhile, CRC cells proliferation and gp130 and p-STAT3 expression were evaluated. In the end, the effect of EBI3 on the tumor growth in vivo was investigated by subcutaneous injecting C26 cells containing EBI3 blocking peptide and their corresponding control cells subcutaneously into BALB/c mice. Here, we demonstrated evidence that EBI3 assist the tumor escape immune surveillance in CRC by mediating a bidirectional reciprocal-regulation STAT3 signaling pathway to induce antitumor CTL response and suppress tumor growth.

## 2. Materials and Methods

### 2.1. Subjects

Subjects were procured from 50 CRC patients who had undergone large bowel resection at the Department of Surgery of Fifth People's Hospital of Dongguan and Houjie Hospital Affiliated to Guangdong Medical University and received no preoperative adjuvant therapy such as radiotherapy or chemotherapy. Subjects consisted of 30 male cases and 20 female cases, aged 32 to 67, with the median patient age of 46 years old. Both cancer and adjacent nontumorous mucosa tissues (named normal tissues, distance from the tumor tissue more than 5 cm) were obtained from operative specimens and stored in −80°C until use. According to the WHO grading system, 30 patients were classified as well and moderately differentiated; other 30 patients were defined as poorly differentiated and undifferentiated. Informed consent was obtained from all study subjects, and the studies were approved by the Internal Review Board of Human Assurance Committee at Fifth People's Hospital of Dongguan and the Guangdong Medical University.

### 2.2. Immunohistochemistry (IHC)

**Figure 1:**
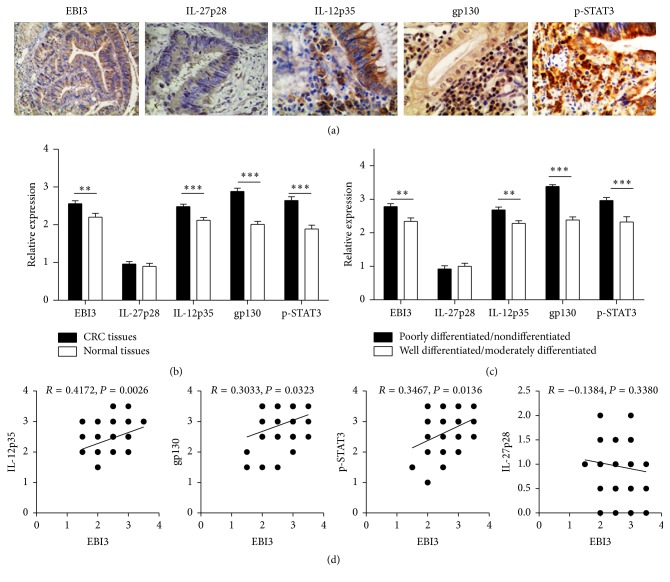
Quantitative analysis of EBI3, IL-27p28, IL-12p35, gp130, and p-STAT3 expression in CRC tissues by immunohistochemistry. (a) Representative images for the immunohistochemical staining of EBI3, IL-27p28, IL-12p35, gp130, and p-STAT3 in CRC tissues (400x). (b and c) Bar graphic figures showing the relative expression levels of EBI3, IL-27p28, IL-12p35, gp130, and p-STAT3 assessed in all tissues. (d) Results for correlation analysis of EBI3 with IL-27p28, IL-12p35, gp130, and p-STAT3 in CRC tissues. ^*∗∗*^ *P* < 0.01; ^*∗∗∗*^ *P* < 0.001.

Tissue sections (4 *μ*M) were prepared from paraffin blocks and then subjected to Hematoxylin and Eosin (HE) staining, as previously reported [13]. For immunostaining, antigenic epitopes were next retrieved by heating for 5 min in 10 mmol/L citrate buffer (pH 6.0); then the slides were incubated in 3% H_2_O_2_ solution for 10 min at room temperature to block the endogenous peroxidase activity. The slides were then first incubated with antibodies against EBI3 (sc-32868, Santa Cruz Biotechnology, Inc.), IL-12p35 (sc-7925, Santa Cruz Biotechnology, Inc.), IL-27p28 (sc-27487, Santa Cruz Biotechnology, Inc.), gp130 (sc-655, Santa Cruz Biotechnology, Inc.), and p-STAT3 (sc-8001-R, Santa Cruz Biotechnology, Inc.) for 30 min at room temperature, followed by HRP-conjugated secondary detection antibody and DAB (Enhanced Polymer) (Kit-0015, Maixin Biotech, Fuzhou, China). Immunoreactivities for the EBI3, IL-27p28, and IL-12p35 positive expression were defined by the cytoplasm appearing as brown granules, and the gp130 and p-STAT3 positive expression were defined by the nucleus and/or cytoplasm appearing as brown granules (Figure 1(a)). The score of immunohistochemical sections was assessed by two pathologists in a blinded fashion to the clinical status of the patients as previously reported [13, 14].

### 2.3. Cell Culture and Mice

Isolated CRC cells, TILs, mouse splenic lymphocytes (SLs), mouse bone marrow-derived macrophages (BMDMs), and C26 cells (Institute of Shanghai Cell Biology and Chinese Type Culture Collection, China) were maintained in 1640 RPMI medium and supplemented with 10% fetal bovine serum (FBS) (HyClone, Logan, UT), 100 units/mL penicillin, and 100 mg/mL streptomycin (Invitrogen) at 37°C in a humidified, 5% CO_2_, 95% air atmosphere. Specific pathogen-free male Balb/c (6 weeks old) mice were purchased from Laboratory Animal Center of Southern Medical University, Guangzhou, China. The mice were maintained in a clean rack in the Laboratory Animal Center at Guangdong Medical University, Zhanjiang, China.

### 2.4. Preparation of CRC Cells, TILs, SLs, and BMDMs

CRC subjects from 10 patients (5 well differentiated and 5 poorly differentiated) were transferred to our laboratory immediately after surgical resection and digested mechanically and enzymatically in 1640 RPMI medium (Gibco, New York, USA) containing 1 mg/mL Blend collagenase N (Worthington, Lakewood, UK), 0.1 mg/mL hyaluronidase III (Sigma-Aldrich, Saint Louis, USA), and 20 *μ*g/mL DNase I (Roche, Rotkreuz, Switzerland) for 2 h at 37°C by a cell separation over a 100 *μ*m cell strainer to obtain a single cell suspension. Tumor cells and TILs were then isolated by Ficoll-Hypaque gradient centrifugation (Dakewe, Beijing, China). Adherent tumor cells were used to do subsequent experiments. SLs and BMDMs were isolated from Balb/c mice. Briefly, the spleen was separated from the mice and placed in a 200-mesh stain steel sieve over a culture dish containing RPMI-1640 medium and grounded into small pieces with the plunger of glass syringe. SLs were then isolated by Ficoll-Hypaque gradient centrifugation (Dakewe, Beijing, China). Bone marrow from femurs and tibias of mice was cultured in RPMI1640 supplemented with 10% FBS and 10 ng/mL murine M-CSF for 6 days and used as BMDMs.

### 2.5. CCK-8 Proliferation Assay for CRCs Cell Growth

Human CRC cells (1.0 × 10^3^/well) were plated in 96-well plates and treated with 1 *μ*g/mL EBI3 blocking peptide (sc-26797 P, Santa Cruz Biotechnology, Inc.) for 1 to 5 days. To ensure the specificity of EBI3 blocking, a matched isotype IgG (sc-34665 P, Santa Cruz Biotechnology, Inc.) was employed as negative controls. 10 *μ*L of CCK-8 (Beyotime, China) was added to the cells, and the viability of the cells was measured at 490 nm using an ELISA reader (BioTek, Winooski, VT, USA) according to the manufacturer's instructions.

### 2.6. CFDA Assay for T Cell Proliferation

For mitogenic response assay, TILs (1.0 × 10^5^/well) were labeled with CFDA (Beyotime, China) for 15 min according to the manufacturer's instructions and cocultured with autogenous CRC cells in the presence of 1 *μ*g/mL EBI3 blocking peptide for 10 days, followed by flow cytometry analysis to assess the proliferation ability of TILs by staining surface markers CD3. SLs (5.0 × 10^5^/well) were cocultured with or without the indicated doses of EBI3 blocking peptide in the presence of ConA (1 *μ*g/mL) in 96-well culture plates for 3 days for mitogenic response assay as described previously. The gating strategy is described in detail in Figure S1 (see Supplementary Material available online at http://dx.doi.org/10.1155/2016/3214105). The CFDA intensity of gated cells was measured through a 518 nm filter (FL1). The data were analyzed using Flowjo.7.6.1 software (Tree Star, Ashland, OR, USA).

### 2.7. Flow Cytometry (FCM)

For assessing EBI3, IL-12p35, IL-27p28, gp130, p-STAT3, Granzyme B, and IFN-*γ* producing TILs, 5 × 10^5^ TILs were cocultured with autogenous CRC cells in the presence or absence of EBI3 blocking peptide as described previously. The gating strategy is described in detail in Figure S1. The suspension cells were then transferred into 5 mL polystyrene round bottom tubes for flow cytometry analysis of surface markers CD45, CD3, CD8, EBI3, gp130, IL-12p35, and IL-27p28 expression and intracellular cytokine Granzyme B and IFN-*γ* expression and p-STAT3 expression.

For assessing IFN-*γ* producing SLs, BMDMs (4 × 10^4^ cells/well) were seeded at 96-well plate and pretreated with or without EBI3 blocking peptide for 10 min and then stimulated with LPS for additional 1 day. Culture supernatants were used for SLs culture and after 3 days' culture, IFN-*γ* producing SLs were analyzed as described [13, 15, 16]. To ensure the specificity of immune staining, a matched isotype IgG was employed as negative controls. Direct ICS was used for measuring T cell producing cytokines without in vitro antigen stimulation, as we recently described [13, 15, 16]. The BD FACSCalibur II (San Jose, CA, USA) platform was used to acquire data. All data were analyzed by using the Flowjo.7.6.1 software (Tree Star, Ashland, OR, USA) as instructed.

### 2.8. Enzyme Linked Immunosorbent Assay (ELISA)

Precoated LEGEND MAX Human IL-27 ELISA kit (BioLegend, San Diego, CA, USA) and human IL-35 ELISA kit (BlueGene, Shanghai, China) were employed for analysis of IL-27 and IL-35 levels in the cell culture supernatants according to the manufacturer's instructions.

### 2.9. Western Blot Analysis

Western blot analysis was performed to determine the expression levels of gp130 and p-STAT3 in CRC cells. Cells were harvested and washed with cold 1x PBS and lysed with RIPA lysis buffer (Beyotime, China) containing 0.5 M DTT, 0.1 M PMSF, and 20x phosphatase inhibitor for 30 min on ice and then centrifuged at 12,000 ×g for 15 min at 4°C. Protein contents were quantified with BCA Protein Assay Kit (Beyotime, China). Equal amounts (30 *μ*g) of protein samples were subjected to 10% SDS-PAGE electrophoresis and transferred on to polyvinylidene fluoride (PVDF) membranes (Millipore, Massachusetts, USA). The membranes were blocked in 5% nonfat milk and incubated with primary antibodies, followed by incubation with secondary antibodies conjugated with horseradish peroxidase (HRP). Immunoblots were visualized by enhanced chemiluminescence detection reagents (Millipore, Massachusetts, USA), with *β*-actin as a loading control.

### 2.10. Tumor Xenograft Models

C26 cells (5 × 10^6^ cells/100 *μ*L) in the presence of 10 *μ*g/mL EBI3 blocking peptide were subcutaneously injected into the flank of the male BALB/c mice (purchased from Laboratory Animal Center of Southern Medical University, Guangzhou, China) at the age of 6 weeks, with presence of IgG and PBS as a control. After the injection of cells, the EBI3 blocked tumors were multipoint injected with EBI3 blocking peptide (2 *μ*g/100 *μ*L) every 2 days and the control groups were injected with IgG and PBS. Tumor diameters were measured 3 times a week, and tumor volumes were calculated according to the following formula (length × width^2^ × *π*/6). After 20 days of treatment, the tumors and spleens were harvested, weighted, and fixed in 4% paraformaldehyde for immunohistochemistry. All the animal experiments were performed after obtaining permission from the Institutional Animal Ethics Committee, Guangdong Medical University.

### 2.11. Statistics Analysis

The results are presented as the mean ± standard error of the mean (SEM). All statistical analyses were performed as previously described [13, 15, 16] using the GraphPad Prism version 5.0 software (GraphPad Software Inc., San Diego, CA, USA). Student's *t*-test was employed to compare the differences of measured data, and Pearson correlation was used to measure the degree of dependency between variables. A value of *P* < 0.05 was deemed significant, and values of *P* < 0.01 and *P* < 0.001 were considered as highly significant.

## 3. Results

### 3.1. Associations of EBI3, IL-27p28, IL-12p35, gp130, and p-STAT3 Expression with Clinicopathological Parameters of Colorectal Cancer Tissues

Although our previously study demonstrated that overexpression of EBI3 and IL-12p35 promotes CRC progression [13], the relationship of EBI3, IL-27p28, IL-12p35, gp130, and p-STAT3 expression with clinicopathological parameters of CRC has not previously been reported. Here, we found that EBI3, IL-12p35, gp130, and p-STAT3 were highly expressed in all CRC sections to compare with the low or negative expression of IL-27p28 (Figure 1(a)). For quantitative analysis of EBI3, IL-27p28, IL-12p35, gp130, and p-STAT3 expression levels in different types of colorectal cancer tissues, the sections were next scored for immunoreactive area as detailed earlier based on the intensity of staining. It was interestingly noted that the expression levels for EBI3, IL-12p35, gp130, and p-STAT3 but not IL-27p28 were correlated to the carcinogenesis of tissue and the extent of cell differentiation. The high expression of EBI3, IL-12p35, gp130, and p-STAT3 was detected in CRC tissues, while in normal samples all of them showed the low expression (Figure 1(b)). Of note, poorly or nondifferentiated tumor tissues showed higher levels of EBI3, IL-12p35, gp130, and p-STAT3 expressions than well or moderately differentiated tumor tissues (Figure 1(c)). However, no correlation was detected in terms of clinical stage, age and gender, and tumor size with EBI3, IL-12p35, gp130, p-STAT3, or IL-27p28 expressions (data not shown). For assessing the relationship of EBI3 with IL-27p28, IL-12p35, gp130, and p-STAT3, the Pearson correlation analysis of section's score was also taken among the 50 patients. Interestingly, we noted tissue EBI3 expression was positively correlated to tissue IL-12p35, gp130, and p-STAT3, but not IL-27p28 expression in CRC patients (Figure 1(d)). Altogether, those data suggest that elevated EBI3 may be associated with IL-12p35, gp130, and p-STAT3 to promote CRC progression.

### 3.2. EBI3 Block Promotes Antitumor Cytotoxic T Lymphocytes (CTLs) Response

**Figure 2:**
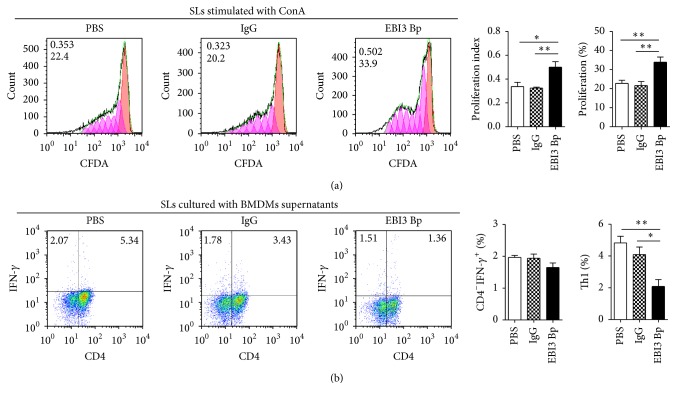
The natural EBI3 signal blocking effect of EBI3 blocking peptide. (a) Mouse splenic lymphocytes (SLs) were isolated from Balb/c mice, SLs were cocultured with or without the indicated doses of EBI3 blocking peptide (EBI3 Bp) in the presence of ConA (1 *μ*g/mL), and SLs proliferation were detected by CFDA assay. (b) Bone marrow from femurs and tibias of Balb/c mice was cultured in RPMI1640 supplemented with 10% FBS and 10 ng/ml murine M-CSF for 6 days and used as mouse bone marrow-derived macrophages (BMDMs). BMDMs pretreated with or without EBI3 Bp for 10 min and then stimulated with LPS for additional 1 day. Culture supernatants were used for SLs culture and after 3-day culture, IFN-*γ* producing SLs were analyzed by FCM. ^*∗*^ *P* < 0.05; ^*∗∗*^ *P* < 0.01.

**Figure 3:**
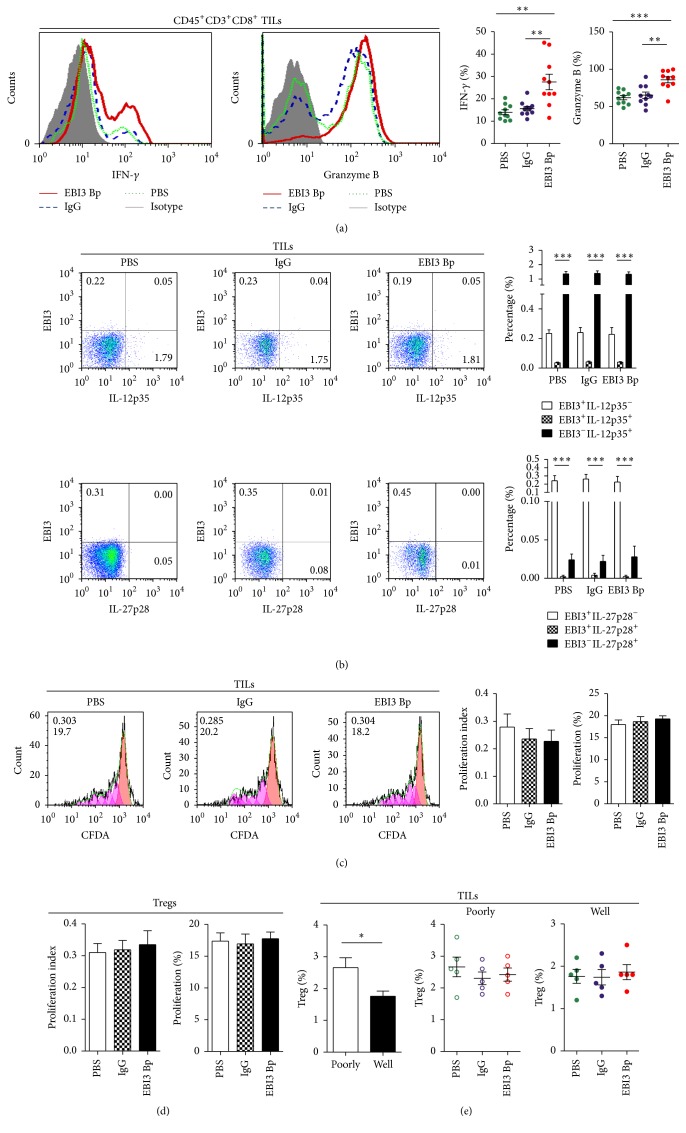
Assessing the role of EBI3 blocking in antitumor CTL response by flow cytometry. (a) Representative images for the Granzyme B and IFN-*γ* production on CD45^+^CD3^+^CD8^+^ TILs after being cocultured with CRC cells stimulated with EBI3 Bp for 3 days, an isotype IgG, and PBS for control. Scatter plot graph showing the percentage of Granzyme B and IFN-*γ* production on CD45^+^CD3^+^CD8^+^ TILs. (b) Representative images for EBI3, IL-27p28, and IL-12p35 expression on CD45^+^CD3^+^ TILs after being cocultured with CRC cells stimulated with EBI3 Bp, IgG, and PBS by flow cytometry. (c) Representative images for CD45^+^CD3^+^CD8^+^ TILs cell proliferation after being cocultured with CRC cells stimulated with EBI3 Bp, IgG, and PBS were detected by CFDA assay. The value (inside) for the percentage of cells that divided at least once (top left corner) and the average number of cell divisions (bottom left corner) are indicated for each groups. (d) Treg cell proliferation after being cocultured with CRC cells stimulated with EBI3 Bp, IgG, and PBS was detected by CFDA assay. (e) Treg cell content in TILs from poorly and well differentiated tumor tissues was detected by FCM after being cocultured with CRC cells stimulated with EBI3 Bp, IgG, and PBS. ^*∗*^ *P* < 0.05; ^*∗∗*^ *P* < 0.01; ^*∗∗∗*^ *P* < 0.001.

In order to explore the mechanism of EBI3 in promoting CRC progression, a EBI3 blocking peptide (named EBI3 Bp), which showed the natural EBI3 signal blocking effect, whether in IL-35-mediated T cell proliferation restricted (Figure 2(a)) or IL-27-mediated Th1 cells differentiation (Figure 2(b)), was used to evaluate the role of EBI3 in tumor microenvironment. Therefore, isolated CRC cells were stimulated by EBI3 Bp before to coculture with autologous TILs as described. Then, functionally active CTLs, Granzyme B and IFN-*γ* production on CD8^+^ TILs, number of EBI3^+/−^IL-12p35^+/−^ or EBI3^+/−^IL-27p28^+/−^ TILs, and cell proliferation ability of TILs and Tregs were detected by FCM. The results showed that Granzyme B^+^ CTLs and IFN-*γ* ^+^ CTLs were increased on EBI3 Bp stimulated CRC cells when cocultured with autologous TILs (Figure 3(a)). Of note, over 98% TILs were EBI3, IL-12p35 double negative (EBI3^−^IL-12p35^−^) phenotype, or EBI3^−^IL-27p28^−^ phenotype cells, whether with or without EBI3 Bp stimulated (Figure 3(b)). Particularly, EBI3^+^IL-12p35^+^ and EBI3^+^IL-27p28^+^ phenotype TILs were undetectable in this study (Figure 3(b)), although IL-35 production Tregs were observed on inflammatory site, but from human nontumor disease [8] or an IL-35 reporter murine models of human cancer [17]. It is now clear that Tregs play a critical role in suppressing antitumor immunity by suppressing CTLs [18]. Therefore, number and cell proliferation ability of TILs and Tregs were evaluated. Obviously, EBI3 Bp stimulated CRCs could not promote or inhibit TILs and Tregs proliferation (Figures 3(c), 3(d), and 3(e)), whether the CRCs are from poorly differentiated or well differentiated tumor tissues, even though they showed different content of Treg cells (Figure 3(e)). Together, these results suggest that EBI3 maybe does not promote Tregs proliferation or mediate TILs proliferation to inhibit CTLs response in tumor microenvironment.

### 3.3. EBI3 Block Inhibits CRC Cell Proliferation and Tumor Growth

**Figure 4:**
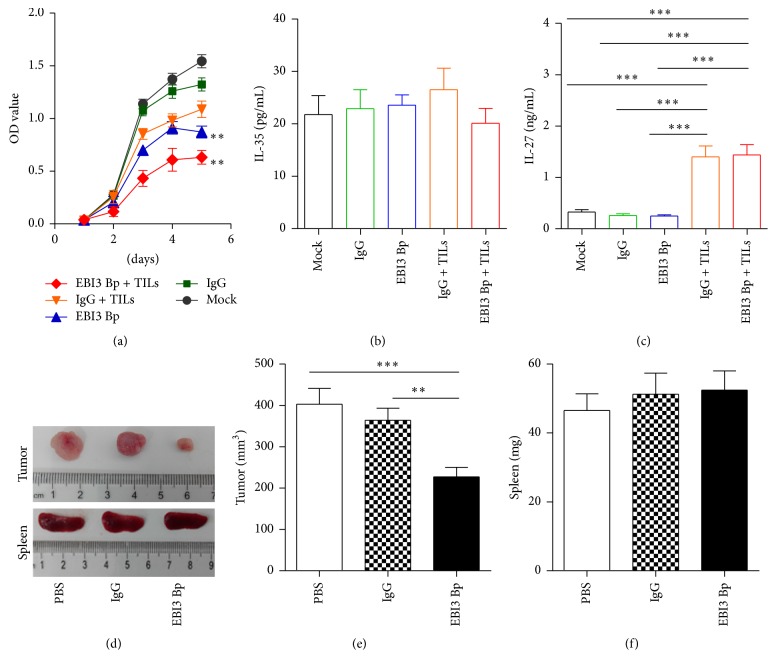
Assessing the role of EBI3 blocking in CRC cell proliferation and tumor growth in vitro and in vivo. (a) CRC cell proliferation after stimulation with EBI3 Bp or coculture with autologous TILs was detected by CCK-8, an isotype IgG, and PBS for control. (b) Supernatants IL-35 levels were detected by ELISA. (c) Supernatants IL-27 levels were detected by ELISA. (d) Representative images of the tumors and spleens at the endpoint from tumor xenograft models. (e) Tumor volumes were measured at the endpoint. (f) Spleens weight was measured at the endpoint. Significant decrease in tumor size but not spleens weight was observed in mice treated with EBI3 Bp as compared to those of control mice. ^*∗∗*^ *P* < 0.01; ^*∗∗∗*^ *P* < 0.001.

Besides inhibiting CTLs response, the role of EBI3 in CRC cell proliferation was evaluated by the EBI3 Bp, as above described. The results showed that EBI3 Bp effectively inhibited CRC cells proliferation as a tumor suppressor (Figure 4(a)). Of note, EBI3 Bp displayed stronger capacity to inhibit CRC cell proliferation when cocultured with autologous TILs (Figure 4(a)). Our previous report had shown EBI3 was high expression on CRC cell membrane and cytoplasm [13] and seen in Figure 1(a). EBI3 can form a heterodimer with IL-27p28 and IL-12p35 subunit to build IL-27 and IL-35, respectively. Therefore, IL-27 and IL-35 levels in the cell culture supernatants were detected by ELISA. Results showed that IL-35 but not IL-27 was obviously expressed on CRCs cell culture supernatants (Figures 4(b) and 4(c)). Interestingly, IL-35 levels on CRCs cell culture supernatants were not affected by the EBI3 Bp stimulation, even though cocultured with autologous TILs (Figure 4(b)). However, only a small amount of IL-27 can be detected when cocultured with autologous TILs (Figure 4(c)). In addition, the effect of EBI3 on the CRC metabolism in vivo was investigated by injecting C26 cells containing EBI3 Bp and their corresponding control cells subcutaneously into BALB/c mice. Tumor sizes were measured every 7 days. C26 cells generated the mean tumor volume of 402.8 mm^3^, while EBI3 blocked C26 cells generated a reduced tumor volume of 227.3 mm^3^ at 20 days (Figures 4(d) and 4(e)). It is worth noting that the weight of spleen was not significantly different in this process (Figures 4(d) and 4(f)).

### 3.4. EBI3 Regulates the Tumor Growth and Antitumor Cytotoxic T Lymphocyte Response by Bidirectional Reciprocal-Regulation STAT3 Signaling Pathway

**Figure 5:**
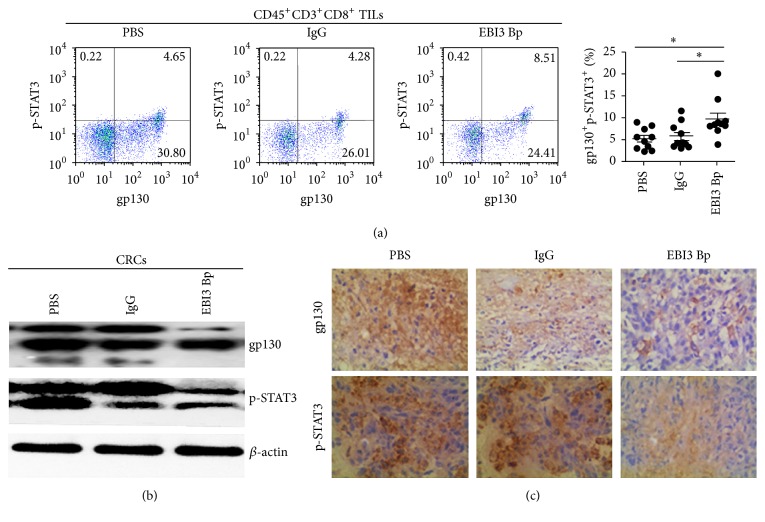
EBI3 regulates the tumor growth and antitumor cytotoxic T lymphocyte response by bidirectional reciprocal-regulation STAT3 signaling pathway. (a) Representative images for the gp130 and p-STAT3 expression on CD45^+^CD3^+^CD8^+^ TILs after being cocultured with CRC cells stimulated with EBI3 Bp for 6 h, an isotype IgG, and PBS for control. Scatter plot graph showing the percentage of gp130^+^p-STAT3^+^ T cells on CD45^+^CD3^+^CD8^+^ TILs. (b) The expression level of gp130 and p-STAT3 was detected by Western blot analysis. (c) gp130 and p-STAT3 in tumor of xenograft models were detected by immunohistochemistry. Low expression of gp130 and p-STAT3 was observed in mice treated with EBI3 Bp as compared to those of control mice. ^*∗*^ *P* < 0.05.

It has been shown that EBI3 via receptor gp130 can induce the activation of STAT3 in NK cell [19], liver pDCs [20], and CD8^+^ T cells [1]. Therefore, p-STAT3 expression by TILs, CRC cells, and tumor after EBI3 block were analyzed by Western blotting, FCM, and IHC, respectively. Results showed that gp130 was constitutively expressed by CD45^+^CD3^+^CD8^+^ TILs and CRCs (Figures 5(a) and 5(b)). It is worth noting that EBI3 block was unable to reduce the expression of gp130 on CD45^+^CD3^+^CD8^+^TILs (date no shown) but able to reduce gp130 expression on CRCs or C26 cells generated tumor (Figures 5(b) and 5(c)). In line with the above results, the expression level of p-STAT3 was reduced on EBI3 blocked CRCs and tumor generated from EBI3 blocked C26 cells (Figures 5(b) and 5(c)). However, we observed a significant increase in STAT3 phosphorylation on CD45^+^CD3^+^CD8^+^ TILs at 6 h after EBI3 blocked peptides stimulation (Figure 5(a)). The above studies suggested EBI3 regulates the tumor growth and antitumor cytotoxic T lymphocyte response by bidirectional reciprocal-regulation STAT3 signaling pathway.

## 4. Discussion

In the present study, we demonstrated evidence indicating that EBI3, IL-12p35, gp130, and p-STAT3 aberrant expression associated with CRC progression. Particularly, EBI3 blocking promoted tumor infiltrating Granzyme B^+^ CTLs and IFN-*γ* ^+^ CTLs production and restrained CRC cell proliferation through bidirectional reciprocal-regulation STAT3 signaling pathway. These findings uncovered a previously unknown mechanism for EBI3 promoting CRC progression associated with IL-12p35, which may have significant implications in terms of diagnosis and treatment of CRC patients in clinical settings.

EBI3 was associated with several human malignancies and detected in EBV-associated tumors, nasopharyngeal carcinoma (NPC), and Hodgkin lymphoma (HL) to inhibit an effective antitumor, independently of its association to IL-27p28 [21, 22]. However, EBI3 association to IL-27p28 enhanced IFN-*γ* production and natural killer (NK) cell activities in a mature T cell-defective condition to produce an effective antitumor for human oesophageal carcinoma cells [23]. Furthermore, suppressed EBI3 expression inhibits lung cancer cell proliferation whereas induction of exogenous EBI3 conferred growth-promoting activity [24]. In the past decade, IL-27 has attracted considerable interest as a potent antitumor cytokine via CD8^+^ T cell-dependent tumor rejection [25], limiting the inducible Treg (iTreg) or Treg population [26, 27], enhancing NK cell response [28], and inhibiting angiogenesis and tumor cell proliferation [7, 29]. Of note, our previously study showed EBI3 association with IL-12p35 to promote CRC progression may recruit Treg cells and other immunosuppressive cells into the tumor microenvironment [13], but the exact mechanisms are not fully understood. Here, we further demonstrated EBI3 not only promoted CRC cell proliferation through the gp130/Stat3 axis, but also restrained tumor infiltrating Granzyme B^+^ CTLs and IFN-*γ* ^+^ CTLs production to promote tumor growth in CRC by EBI3 blocking peptide in vitro and in vivo. Interestingly, we also found not only IL-12p35 but also gp130 and p-STAT3 were noted to be positively correlated to EBI3 in CRC tissues. Isolated CRC cells from patients were stimulated with EBI3 blocking peptide before to coculture with autologous TILs. Results found EBI3 blocking to promote Granzyme B and IFN-*γ* production on CD8^+^ TILs but did not affect T cell proliferation or EBI3^+^, IL-12p35^+^, and IL-27p28^+^ T cell production, which indicated EBI3 has nothing to do with iTreg in assisting CRC cells escape immune surveillance.

Furthermore, EBI3 blocking suppressed CRC cell proliferation through the gp130/Stat3 pathway, which mediated cell survival in various tumor, such as gastric cancer [30], breast cancer [31], hepatocellular carcinoma [32], and also CRC [33, 34]. Meanwhile, our results suggested not only secretion of IL-11 by TGF-*β*-stimulated cancer-associated fibroblasts (CAFs) [34] but also EBI3 derived from CRC cells triggers gp130/STAT3 signaling to promote tumor growth. Consistent with this hypothesis, the effect of EBI3 on the CRC metabolism in BALB/c mice was investigated by injecting C26 cells in the presence of EBI3 blocking peptide. In line with the above results, EBI3 blocked suppressed tumor growth with a low expression of gp130 and p-STAT3. Indeed, intravenous injection of the B16-F10 cell line in EBI-3^−/−^ recipient mice resulted in a significant reduction of lung tumor metastasis [35]. In contrast, BALB/c mice inoculated with EBI3 overexpressed C26 cells developed tumors and the tumors continued to grow [36]. In consistent with published data, we noted that EBI3 in the tumor microenvironment are likely to be a prerequisite to ensure CRC progression. It has been shown that EBI3 via receptor gp130 can induce the activation of STAT3 in NK cell [19], liver pDCs [20], and CD8^+^ T cells [1]. Here, the expression level of p-STAT3 was reduced on EBI3 blocked CRCs and tumor generated from EBI3 blocked C26 cells but increased on CD45^+^CD3^+^CD8^+^TILs at 6 h after EBI3 blocked peptides stimulation, suggesting EBI3 regulates the tumor growth and antitumor cytotoxic T lymphocyte response by bidirectional reciprocal-regulation STAT3 signaling pathway.

In summary, we demonstrated evidence that EBI3 associated with IL-12p35, gp130, and p-STAT3 are highly expressed in CRC cells to inhibit antitumor CTL response and promote tumor growth in CRC.

## Supplementary Material

The Supplementary Material is the gating strategy for the detection of various indicators by Flow cytometry analysis.
