# Corrigendum to “Epstein-Barr Virus-Induced Gene 3 (EBI3) Blocking Leads to Induce Antitumor Cytotoxic T Lymphocyte Response and Suppress Tumor Growth in Colorectal Cancer by Bidirectional Reciprocal-Regulation STAT3 Signaling Pathway”

**DOI:** 10.1155/2017/2917134

**Published:** 2017-07-27

**Authors:** Yanfang Liang, Qianqian Chen, Wenjing Du, Can Chen, Feifei Li, Jingying Yang, Jianyu Peng, Dongping Kang, Bihua Lin, Xingxing Chai, Keyuan Zhou, Jincheng Zeng

**Affiliations:** ^1^Department of Pathology, Dongguan Hospital, Medical College of Jinan University, The Fifth People's Hospital of Dongguan, Dongguan 523905, China; ^2^Guangdong Provincial Key Laboratory of Medical Molecular Diagnostics, Guangdong Medical University, Dongguan 523808, China; ^3^Department of Integrative Medicine, Huashan Hospital, Fudan University, Shanghai 200040, China

In the article titled “Epstein-Barr Virus-Induced Gene 3 (EBI3) Blocking Leads to Induce Antitumor Cytotoxic T Lymphocyte Response and Suppress Tumor Growth in Colorectal Cancer by Bidirectional Reciprocal-Regulation STAT3 Signaling Pathway” [1], there were errors in the title, Introduction, Materials and Methods, and the legend of Figure  2, which should be corrected as follows:

The title should be corrected to “Epstein-Barr Virus-Induced Gene 3 (EBI3) Blocking Leads to Induction of Antitumor Cytotoxic T Lymphocyte Response and Suppression of Tumor Growth in Colorectal Cancer by Bidirectional Reciprocal-Regulation STAT3 Signaling Pathway.”

In the last sentence of the Introduction, “suppress” should be corrected to “promote.”

In the Materials and Methods, in Section  2.3 “Balb/c” should be corrected to “BALB/c,” in Section  2.4 “Balb/c” should be corrected to “BALB/c” and “RPMI1640” should be corrected to “RPMI 1640,” and in Section  2.9 “1x PBS” should be corrected to “1 × PBS.”

In the legend of Figure  2 “Balb/c” should be corrected to “BALB/c” and “RPMI1640” to “RPMI 1640.”
