# Supplementary material for: Epstein-Barr Virus-Induced Gene 3 (EBI3) Blocking Leads to Induce Antitumor Cytotoxic T Lymphocyte Response and Suppress Tumor Growth in Colorectal Cancer by Bidirectional Reciprocal-Regulation STAT3 Signaling Pathway
Source: Mediators Inflamm. 2016 May 10;2016:3214105. doi: 10.1155/2016/3214105 (PMC4877478; doi:10.1155/2016/3214105)
Supplement: Supplementary file 1 — The Supplementary Material is the gating strategy for the detection of various indicators by Flow cytometry analysis. [file 3214105.f1.zip › Caption of Figure S1.docx]

Figure S1: Gating strategy. TILs (P1) were gated on FSC and SSC dot plots. CD45^+^ TILs (P2) were gated from P1. Then, CD3^+^ T cells (P3), CD4^+^CD25^+^ T cells (P4), CD3^+^CD8^+^ T cells (P5) were gated from P2. The subsets of EBI3^+^IL-12p35^+^ T cells, EBI3^+^IL-27p28^+^ T cells were detected from gate P3. Treg cells counts and proliferation of Treg cells were detected from gate P4. the gp130 and p-STAT3 expression, the Granzyme B and IFN-γ production, and proliferation of CD45^+^CD3^+^CD8^+^ TILs were detected from gate P5.
