# Supplementary figures and images for: Epstein-Barr Virus-Induced Gene 3 (EBI3) Blocking Leads to Induce Antitumor Cytotoxic T Lymphocyte Response and Suppress Tumor Growth in Colorectal Cancer by Bidirectional Reciprocal-Regulation STAT3 Signaling Pathway
Source: Mediators Inflamm. 2016 May 10;2016:3214105. doi: 10.1155/2016/3214105 (PMC4877478; doi:10.1155/2016/3214105)

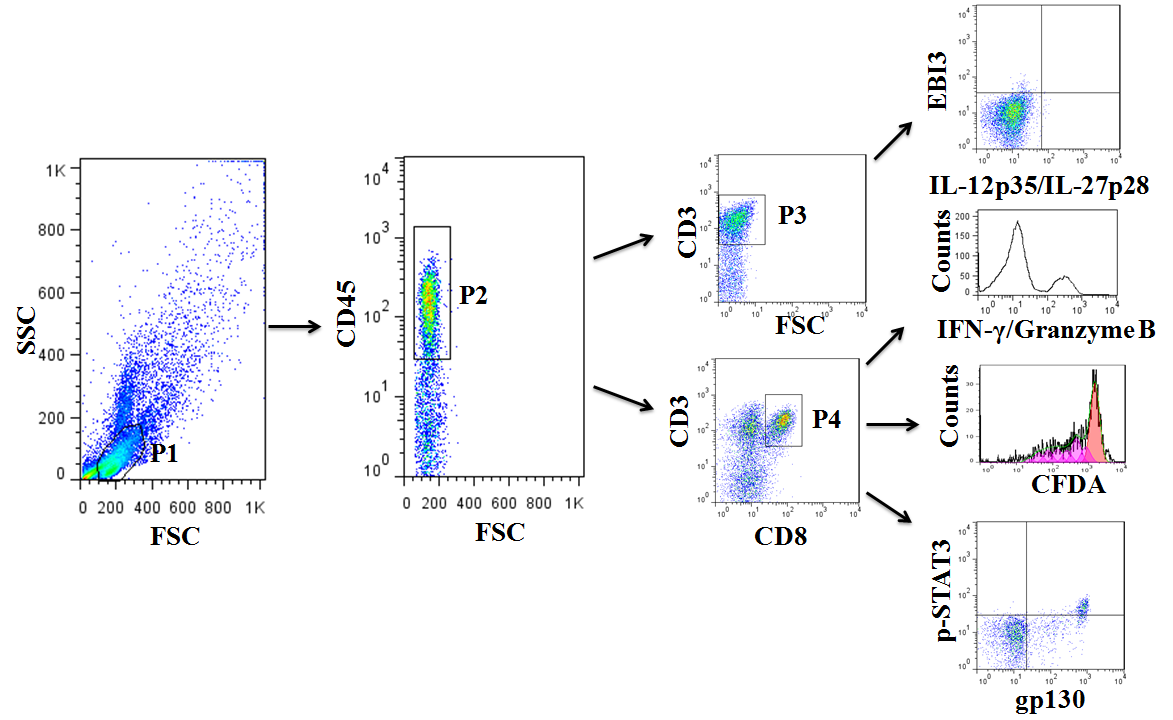

Supplement: Supplementary file 1 — The Supplementary Material is the gating strategy for the detection of various indicators by Flow cytometry analysis. [file 3214105.f1.zip › Figure S1.tif]
